# The disambiguation of people names in biological collections

**DOI:** 10.3897/BDJ.10.e86089

**Published:** 2022-10-10

**Authors:** Quentin Groom, Christian Bräuchler, Robert W. N. Cubey, Mathias Dillen, Pieter Huybrechts, Nicole Kearney, Niels Klazenga, Siobhan Leachman, Deborah L Paul, Heather Rogers, Joaquim Santos, David Peter Shorthouse, Alison Vaughan, Sabine von Mering, Elspeth M Haston

**Affiliations:** 1 Meise Botanic Garden, Meise, Belgium Meise Botanic Garden Meise Belgium; 2 Naturhistorisches Museum Wien, Wien, Austria Naturhistorisches Museum Wien Wien Austria; 3 Royal Botanic Garden Edinburgh, Edinburgh, United Kingdom Royal Botanic Garden Edinburgh Edinburgh United Kingdom; 4 Biodiversity Heritage Library (BHL) Australia, Melbourne, Australia Biodiversity Heritage Library (BHL) Australia Melbourne Australia; 5 Royal Botanic Gardens Victoria, Melbourne, Australia Royal Botanic Gardens Victoria Melbourne Australia; 6 Independent Researcher, Wellington, New Zealand Independent Researcher Wellington New Zealand; 7 University of Illinois, Champaign, United States of America University of Illinois Champaign United States of America; 8 Florida State University, Tallahassee, United States of America Florida State University Tallahassee United States of America; 9 McGill University, Montreal, Canada McGill University Montreal Canada; 10 Centre for Functional Ecology, Department of Life Sciences, University of Coimbra, Coimbra, Portugal Centre for Functional Ecology, Department of Life Sciences, University of Coimbra Coimbra Portugal; 11 Agriculture & Agri-Food Canada, Ottawa, Canada Agriculture & Agri-Food Canada Ottawa Canada; 12 Museum für Naturkunde, Leibniz Institute for Evolution and Biodiversity Science, Berlin, Germany Museum für Naturkunde, Leibniz Institute for Evolution and Biodiversity Science Berlin Germany

**Keywords:** authority file, attribution, biography, linked open data, Wikidata

## Abstract

Scientific collections have been built by people. For hundreds of years, people have collected, studied, identified, preserved, documented and curated collection specimens. Understanding who those people are is of interest to historians, but much more can be made of these data by other stakeholders once they have been linked to the people’s identities and their biographies. Knowing who people are helps us attribute work correctly, validate data and understand the scientific contribution of people and institutions. We can evaluate the work they have done, the interests they have, the places they have worked and what they have created from the specimens they have collected. The problem is that all we know about most of the people associated with collections are their names written on specimens. Disambiguating these people is the challenge that this paper addresses. Disambiguation of people often proves difficult in isolation and can result in staff or researchers independently trying to determine the identity of specific individuals over and over again. By sharing biographical data and building an open, collectively maintained dataset with shared knowledge, expertise and resources, it is possible to collectively deduce the identities of individuals, aggregate biographical information for each person, reduce duplication of effort and share the information locally and globally. The authors of this paper aspire to disambiguate all person names efficiently and fully in all their variations across the entirety of the biological sciences, starting with collections. Towards that vision, this paper has three key aims: to improve the linking, validation, enhancement and valorisation of person-related information within and between collections, databases and publications; to suggest good practice for identifying people involved in biological collections; and to promote coordination amongst all stakeholders, including individuals, natural history collections, institutions, learned societies, government agencies and data aggregators.

## Introduction

Biological collections contain a wealth of information on the occurrence of organisms and the people who collected them. As scientific objects of human endeavour, biological specimens rely on people to collect, curate, identify, image and examine them. As records of the existence of taxa at particular points in time and place, they rely on accurate information on the movements and activities of people in order to maximise their fitness-for-purpose. People are central to biological collections and accurate data about the people who collect and care for them is an essential part of the scientific record.

The many and varied traces each of us leave behind in the course of our lives provide clues to our identities, our activities and our movements (Groom et al. 2020). People are identified in writing to give them credit for their work, to provide authority to the statements they make and to link them to their corpus and networks. Publications, field notes, specimen labels, databases, collection registries and logs of research ships all contain the names of people undertaking technical and scientific pursuits. We also leave traces of our activities beyond these pursuits that can help refine the accuracy and repeatability of our science. Birth certificates, death certificates, immigration records, passports, census data, family trees, correspondence, affiliations with institutions and societies and other documents and statements about our lives are all circumscribed within the boundaries of time and space. The names of people are important aids in the discovery of related information. They are also vital entry points into the rich, emotive narratives of human activity, which play a powerful role in relaying the urgency and importance of our scientific work (Weil 2007, Constant and Roberts 2017).

Biographical information about individuals can be used to validate scientific data associated with their work and that of their collaborators. Collecting dates and localities can be verified from personal details such as birth, death and marriage dates, places of residence and employment, personal interests and travels. By using biographical information, redundant digitisation or analytical efforts on related biodiversity data records can be avoided and incorrect or inaccurate information rectified. Missing pieces of data may be inferred, which become easier as more records can be linked through common identifiers. Thus information, such as the key dates in a person’s life, the places they visit and the people they know, are important.

Data about people are widely used in historical research, but these data have many other uses in every scientific discipline. Here, we specifically focus on people associated with biological collections. These are mainly people who collect, curate, identify and analyse biological specimens, but they are also people who name new species, study their ecology, undertake a variety of other research and publish their work. Unambiguously identifying people also makes it easier to quantify their contributions, particularly for those, such as taxonomists, for whom citation indices are not necessarily a useful indicator of output, nor quality (Valdecasas 2011). Furthermore, broadening the evaluation of scientific contributions may help increase the diversity and inclusivity of career evaluations (Bhalla 2019, Woolston 2022).

People are typically identified by their names, whether in full or abbreviated. However, people’s names are not unique identifiers. It is difficult to know whether a single name string refers to one or more people (Finch 2008, Smalheiser and Torvik 2011) or whether multiple different name strings refer to a single individual. In writing, names are rendered as a sequence of characters with variations in format and script that are often a function of cultural practices. Consequently, one person can be referred to by several different versions of their name, none of which is guaranteed to be unique to that individual. To use biographic data for the purposes outlined above, we need to be able to identify individuals unambiguously. In this paper, we use the term ‘disambiguation’ to mean the processes by which we resolve any uncertainty in the use of a person’s name using any and all corroborating information. The end result of the process is a declaration of the shared identity/ies of a person referred to for any unresolved character string for a person name expressed using the breadth of evidence for the assertion, ideally attached to a persistent identifer.

The process of disambiguation is a particular challenge for natural history collections, where data about individuals – and the specimens collected or annotated by them – are often distributed amongst many different collections, publications and databases. In this paper, we focus on people, though we acknowledge that there are other “agents” that potentially perform roles in collections and on specimens, such as the parent organisation or automated machine processes.

Disambiguation is a process that brings multiple benefits. Clarifying the "who" for specimens extends the number of records useful for research by linking them together; it aids data analysis by helping identify duplicate specimens collected by the one person; it helps resolve past and present collector networks (Groom et al. 2014, Meeus et al. 2021); it supplies collections with information they can use to better resolve their holdings and share with other collections (Groom et al. 2020, Güntsch et al. 2021); it facilitates discovery of a given group of experts through time and space; it provides researchers with additional and direct paths to publishing their collecting and identifying expertise-effort; and, lastly, it demonstrates how the work of each individual contributes to the biodiversity knowledge graph (Page 2016).

The widespread and collaborative nature of biological collections necessitates a shared approach to disambiguation that utilises robust data-sharing mechanisms to avoid the duplication of effort or replication of errors. All people, living or deceased, who have contributed to biological collections ought to have a persistent, unique and freely-sharable identifier. This requires a strategy that reaches well beyond the narrowness of the biological sciences through explicit reliance on other, well-recognised platforms and solutions. Although there is some domain specificity required, the disambiguation of person names in biological data requires a battery of generic tools and services that draw upon multiple lines of evidence, terminating in authoritative, stable and canonical representations of identity. We expect these mechanisms to be equally useful for other domains and to further contribute to the concept of linked open data.

This paper provides particular guidance on the disambiguation of people who, being deceased, are unable to disambiguate their own names. Living people have an incentive to maintain their public biographical data in public resources and have a responsibility to do so if they are employed to generate scientific output. If they are providing citable resources to the scientific record, then they should help maintain the integrity of these data, including their own identity. This does not mean they have to share any personal information, but registering and using an Open Researcher and Contributor ID (ORCID) is a simple step each researcher should take to preserve their own scientific legacy and the legacy of the institution that employs them. Disambiguating one's own identify also avoids generating disambiguation work in the future (Groom et al. 2020).

In this paper, we review and propose best practices for the disambiguation of people in collections. We provide strategies that can be used and considerations for the prioritisation of the work. We outline the biographical resources available for disambiguation, the tools for making the process efficient, the options for unique identifiers and the best practices and recommendations for documenting disambiguation, expressing uncertainty and maintaining these data in databases. We provide examples as case studies and detail the pitfalls that can be encountered. Finally, we suggest some of the uses for these data and consider the possibilities of globally disambiguated collections, including the positive feedback loops of the disambiguation process.

## Disambiguation in Society

### Ethical and legal considerations

Any dealings with information about people and their activities require careful consideration of the ethical and legal implications of collecting and sharing personal data. Digital technologies have made it easy to gather extensive information on a large number of people. There have always been moral and legal constraints related to the use of data; however, data on people's activities have long been published in biodiversity literature. The power of digital technologies to process these data has prompted governments to enact legislation to formalise the rules regarding the collection and processing of personal data. In Europe, the General Data Protection Regulation (GDPR) is a notable example (Copas 2019, Staunton et al. 2019, European Data Protection Supervisor (EDPS) 2020). However, even in the absence of legal constraints, we have a moral obligation to be sensitive and conscientious when handling personal data. GDPR establishes various legal bases on which data can be kept and processed. One legal basis is for people to give their consent, but another is “legitimate interest” (European Data Protection Supervisor (EDPS) 2020). In the case of those individuals authoring publications and/or collecting and identifying specimens, there is a clear legitimate interest of scientific collections to collate and share relevant data on these people. GDPR also only applies to living people; however, this does not mean there is not a responsibility to be considerate to living relatives of deceased people. GDPR has additional restrictions for “sensitive data”. These are data such as the racial or ethnic origin of a person, their political opinions, religion or philosophical beliefs. It also includes any genetic or health-related data and biometric data for identification; or data concerning a person’s sex life or sexual orientation. While some of these sensitive data might, on occasion, be useful in disambiguation, the costs in complying with the law and the potential ethical jeopardy would almost certainly outweigh the benefits for collections. Therefore, we recommend that sensitive data are not used by collections for disambiguation and should not be stored in collection databases. There are occasions when such data are in the public domain but, if such data need to be stored for research purposes, they should be done under the supervision of the ethical policy of the research institution. Other personal data of living people, such as email addresses and birth dates are covered under GDPR legislation and may be stored in collection management systems, but they cannot be shared without consideration of the regulations.

Outside of Europe, each jurisdiction has different regulations governing the collection and use of personal data. In many cases, laws around the disclosure of personal information echo the intent of GDPR's legitimate interest; however, to our knowledge, these principles remain untested in relation to natural history specimens and it is beyond the scope of this paper to provide guidance on privacy law. It is important that data managers are cognisant of local legislation and the implications of sharing data beyond legal jurisdictions. In addition to legal protections on the use of personal data, specific codes of ethics have been drawn up for museums (Lewis 2004, International Council of Museums 2017, Macdonald 2018, Rabeler et al. 2019). Yet, it is important to be aware that rapid advances in law and technology have changed the ways collections operate and codes of conduct and institutional policies have not necessarily kept pace. Researchers and institutions need to be vigilant of the changing legal, technical and ethical landscape and change their practices where necessary.

### Cultural considerations

There are many cultural differences that influence how people's names are constructed and used and this makes their disambiguation even more complex. The Anglo-Saxon traditional sequence of title, first name, middle name/s, family name/s, suffix is only one of many ways in which names are constructed (see case studies for examples). Software that is constrained to one cultural norm can lead to the truncation and misspelling of names from other cultures. For example, diacritics and ligatures are partly cultural and, in digital files, are partly based on the age of the database. Before Unicode (i.e. pre-1987), software predominantly used ASCII text that severely limited the available characters; we are still living with the legacy from this period. Variations in the ability of different Collection Management Systems to accommodate diacritics and ligatures can result in multiple versions of an individual's name. This in turn amplifies the need for disambiguation, particularly in databases that have been migrated to newer systems. Simplifications of names due to their encoding can itself lead to mistakes and potentially offend. This is a large subject, but fortunately the W3C organisation has a detailed document on it (Ishida 2011).

### Prejudices & biases

A significant benefit of disambiguating people's names and identities is that it can help give recognition to women in the sciences whose contributions to biodiversity and botanical knowledge have historically been pushed to the margins and under-recognised (Shteir 1996). In a Western European and North American context, socio-cultural barriers devaluing women's knowledge of the natural world resulted in either a lack of adequate contribution or no contribution at all (George 2006, Lindon et al. 2015, Meeker and Szabari 2020). Specifically, the cultural norm where women are identified in reference to their husbands (e.g. Mrs. Joseph Clemens) can make it hard to unambiguously identify women collectors (Fig. 1) (also see case studies below). Moreover, data entry protocols can serve to further obscure their contributions to natural history by making the women findable only via their husband's name (Maroske and Vaughan 2014).

## The informatics landscape of disambiguation

The mass digitisation of specimens and the open sharing of data have completely changed the potential for disambiguation globally. In addition to this, the availability of open editable identifiers has accelerated the processes many fold. Here, we introduce the core elements of the disambiguation landscape: Wikidata, ORCID and Bionomia as a means to link them.

### Wikidata

Wikidata has revolutionised our ability to disambiguate people: it contains only public domain data; it can be edited by anyone; and it is readable by both people and machines. Wikidata is not a primary source for biographical data; indeed, each entity is expected to be referenced to a primary source. Wikidata provides a globally unique and resolvable URL identifer by assigning a "Q identifier" for each person (e.g. the phycologist Josephine Tilden Q20856036). Structured statements describe detailed characteristics of an "Item", such as a person, through community-agreed sets of properties (e.g. the property for "date of death" is P570), values (e.g. 1955-01-12) and supporting evidence – the reference to the primary source (e.g. a URL to an obituary). Statements are created by human volunteers as well as authenticated scripts, known as bots. All data are dedicated to the Public Domain and may be freely accessed through a web interface or through scripted SPARQL queries. Many properties exist for external identifiers (e.g. DOI = P356, ORCID = P496) and, as such, Wikidata is a powerful tool that may be used to store and connect all existing identifiers and to reconcile, broker and resolve entities using their previously disconnected identifier systems.

**Figure 2.**
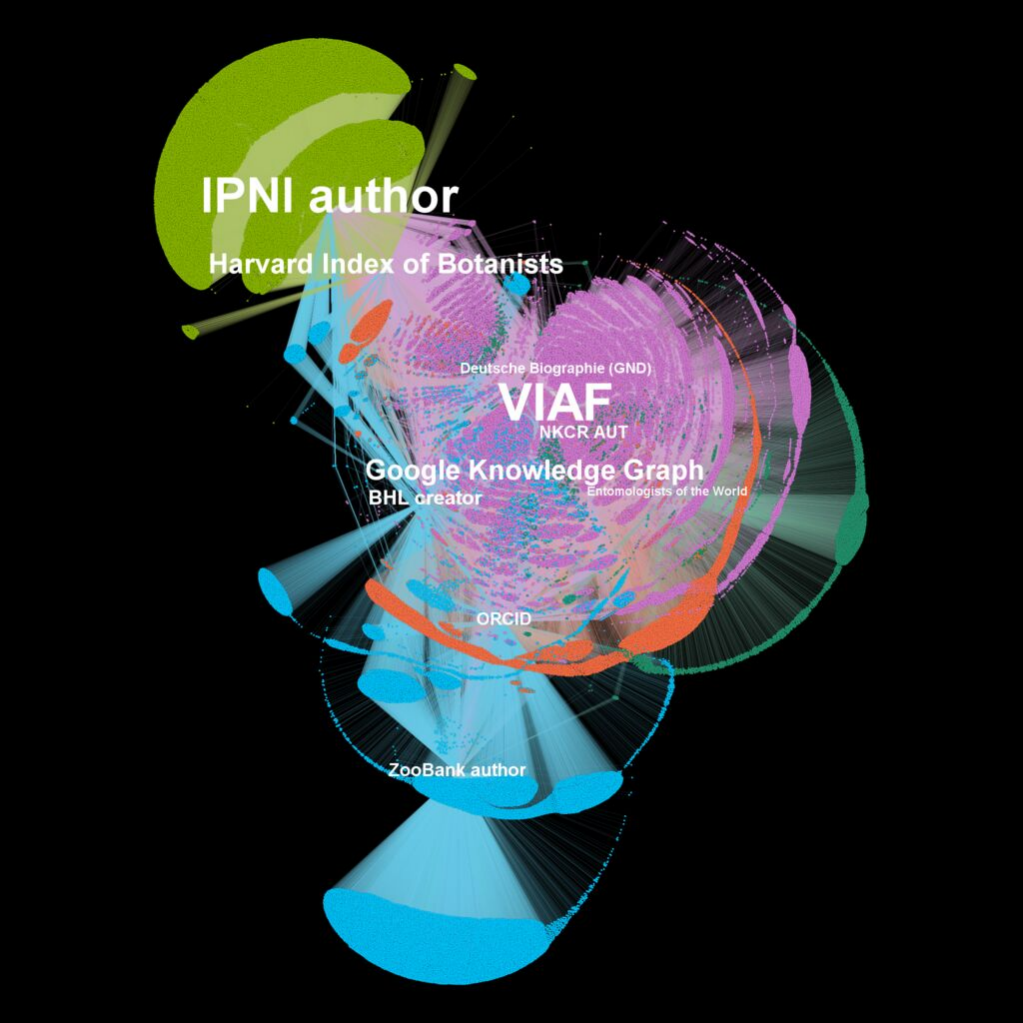
A network of the top twenty most used identifiers for biologists on Wikidata. Ten of the more distinct identifiers are labelled with the font size proportional to the number of linked people. The other identifiers are the Dutch National Thesaurus for Author names, the Center of Warsaw University Library catalogue, International Standard Name Identifier, German National Library ID, Library of Congress authority ID, Bibliothèque nationale de France ID, the identifier for authority control in the French collaborative library catalogue, Freebase ID and WorldCat Identities ID, all of which cluster closely together with the VIAF ID due to the large amount of redundancy across those databases. Lastly, the botanist author abbreviation clusters with the IPNI author because there is a one-to-one relationship between these two identifiers. These data were extracted from Wikidata on 18-02-2022. They were visualised using Gephi (Bastian et al. 2009) using the Yifan Hu layout algorithm (Hu 2005) and the five coloured modules are identified using the algorithm of Blondel et al. (2008).

A Wikidata entry not only provides core biographical details of people, but it also creates a bridge between different sources of authority, such as VIAF and ORCID (Fig. 2). By using Wikidata as a tool for disambiguation and contributing disambiguated data back into it, we can create open public records for people and link them to other resources which bring together all the public information about that person. Traditionally, disambiguation work would have only been documented within a local collection management system available only to local users and few collection management systems have supported capabilities to perform and document such disambiguation work. Now, with Wikidata, important elements of biography can be made available to the whole community. As Fig. 2 illustrates, Wikidata brings together biographical information from the different disciplines of biology. There are some identifiers for specific groups of people, such as botanists, entomologists and zoologists, but it is also clear that there is large overlap between the people in all of these different sources.

Wikidata, like Wikipedia, has the advantage of being community-edited. It is a centralised resource and ensures that data on contributors to collections are openly linked and can be easily enriched and corrected. The notability criteria for the creation of a Wikidata item are significantly lower than that for the creation of a Wikipedia article (see case study below on Winifred Chase); a person is notable for Wikidata if they "*can be described using serious and publicly available references*" (https://www.wikidata.org/wiki/Wikidata:Notability). Wikidata is also a multilingual project allowing editors to contribute in their preferred language.

### ORCID

An ORCID (Open Researcher and Contributor IDentifier) is a globally unique, persistent identifier that is free of charge. It is an essential tool to unambiguously identify researchers when submitting manuscripts for publication, grant applications and other scholarly activities (Haak et al. 2012). ORCID makes its metadata publicly available through an application programming interface at the full discretion of each researcher. ORCID is a global, not-for-profit organisation that is supported by membership fees. It is governed by a Board of Directors with representation from the library, publications and research communities. Unlike Wikidata Q Identifiers, which can be created and edited by anyone, ORCID profiles are established and maintained by the individuals they represent. This means ORCIDs have two major limitations:

1. they cannot be used for people who are unable (i.e. the dead or infirm) or unwilling to create one and
2. they often contain too little information to make them useful for disambiguation; it is possible to create a profile with no information at all and for the owner to make their profile private. A detailed and publicly available profile is the best aid for disambiguation.

### Bionomia

Bionomia is an online, open data curation tool for the disambiguation of collectors and determiners of specimens (Shorthouse 2020). Occurrence records are periodically downloaded from the Global Biodiversity Information Facility (GBIF) then re-indexed and re-organised according to the likelihood that each specimen, as a component of the occurrence, was collected and/or identified by a particular agent. Users of Bionomia then actively verify or reject these candidate, algorithmically-produced assertions. The infrastructure uses a combination of name parsing to split lists of agent name strings into component parts, graph theory algorithm to identify and rank agent name strings and search logic to facilitate human judgement. Participants assert associations between GBIF occurrence records and people as these are represented by their presence in ORCID or Wikidata. As of July 2022, 1,825 participants have either claimed or attributed 22M occurrence records to either an ORCID or a Wikidata Q Identifier, thus persistently linking them to a uniquely identified individual. Collection curators are encouraged to use the data from Bionomia to enrich their collection management system by downloading relational, Frictionless Data packages, incorporating the asserted links, then republishing their data to GBIF with the inclusion of these new links in the Darwin Core fields recordedByID and identifiedByID. GBIF is working with Bionomia to raise awareness and make it easier to bring annotations into collection management systems, similar to other data quality flags.

**Figure 3.**
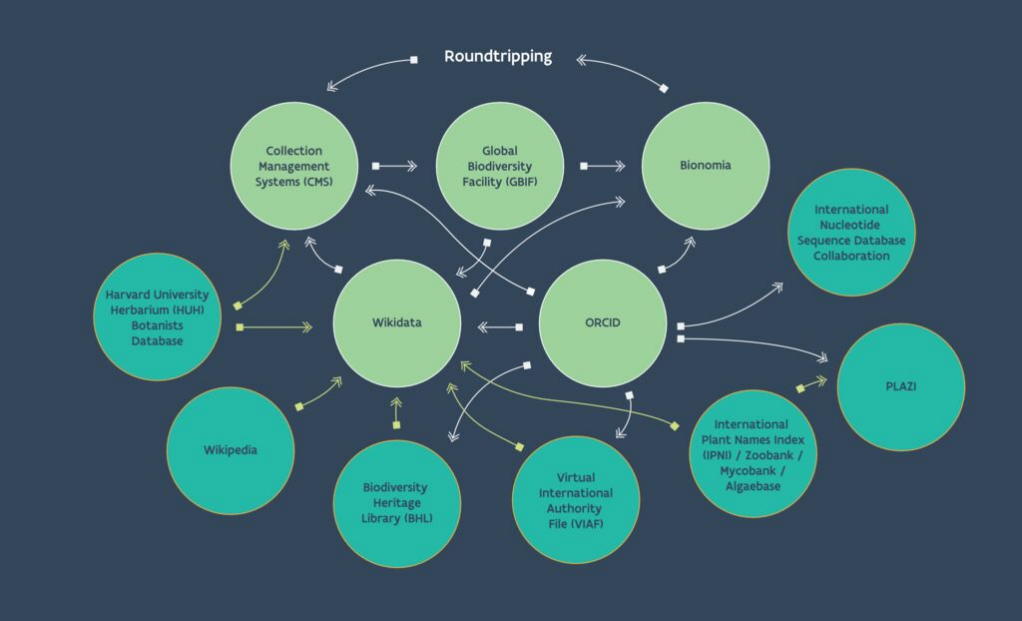
The connections between the core platforms of open disambiguation for natural history collections (light green) and their connections with other important biodiversity informatics platforms (blue). The diagram shows how data flow from collection management systems, through GBIF and is connected in Bionomia to people through Wikidata and ORCID. These new links can then be returned to the system. This data enrichment cycle is referred to as data roundtripping. Other biodiversity informatics platforms facilitate this process by providing biographical data and other information that support disambiguation.

Bionomia is one example of an online platform where person data participate in a roundtrip (Fig. 3). Other domains have also explored data roundtripping, most notably in libraries, archives and art museums (Larsson et al. 2019, Triebel and Scholz 2022, Agenjo-Bullón and Hernández-Carrascal 2020). There are many semi-structured data elements in collection management systems that could benefit from the application of shared, unique identifiers in open data curation environments with roundtrips back to their source. Geographic place names, scientific names and concepts and bibliographic references are prime candidates to help democratise the exchange of metadata and information through unified services that increase efficiencies, deduplicate effort and engender new collaborations (Nicolson et al. 2018, Güntsch et al. 2021).

### Sustainability

The resources and infrastructure of disambiguation need to be sustainable. Wikidata and ORCID have funding strategies and governance models to ensure their longevity. As the museum and herbarium community do not have the funds and capital to replicate these resources within their own domain, there is much value in supporting these common open resources. Bionomia is presently in the start-up, exploratory phase of its development, but is, nonetheless, open by design. It aids disambiguation through stand-alone libraries of code, reusable search algorithms, user-driven exports to digital archives and wholesale downloads that contain annotated links using open standards. Sustainability is less of an issue for Bionomia because it is just a tool, albeit a very useful one, which would eventually become redundant once collections are completely disambiguated.

There is an opportunity now for the community of biological collections to recognise the great value gained from these resources and to engage with them to help steer their development and to contribute data. Ultimately, open informatics resources survive if they are useful to someone and, thereby, give sufficient value to gain investment. We believe this will be the case for these resources and, as more collections join this initiative, the more permanent these resources will be.

## Relevant informatics resources

In additional to the other informatics resources, we mention two others which deserve particular attention, these being the exchange data standards and the tool OpenRefine.

### Exchange standards

Darwin Core and ABCD are the two primary standards for exchanging data on biological specimens (Wieczorek et al. 2012, Holetschek et al. 2012). Recently, Darwin Core added the terms recordedByID and identifiedByID to allow the addition of person identifiers to the terms recordedBy and identifiedBy. Additionally, the latest ABCD release version 3.0 is able to accommodate identifiers for people (Access to Biological Collection Data task group 2019). There are other roles for people involved in the collection, identification and study of specimens. Currently, the People in Biodiversity Data Task Group of the Biodiversity Information Standards organisation are working on an extension to Darwin Core Archive exchange format that will expand the range of options for documenting information about people and their roles. This will include name strings and identifiers for multiple people in ranked order. Nevertheless, even this extension to the standard is not intended to handle multiple identifiers for the same person and the complexities of verbatim name strings, versus canonical names. Such details will still be important in biographical databases and collection management systems.

### OpenRefine

OpenRefine is a browser-based tool for cleaning, transforming and enriching tabular data. OpenRefine allows users to import spreadsheet style data, which can then be reformatted using a simple expression language known as GREL, built to resemble JavaScript. Alternatively, more technical users can also write expressions in Jython (a Java implementation of Python) or Clojure. Importing libraries from the last two languages greatly extends the possibilities for transforming data within OpenRefine, for example, the processing of XML/HTML elements via jsoup. This also allows linkage to web services, whereby matches to potential names can be made and users can select the disambiguated name (van Hooland et al. 2013). OpenRefine is particularly well suited to bulk disambiguation of records, because it can apply decisions across multiple rows and implements several fuzzy matching algorithms for record clustering, including phonetic matching. This is especially useful for terms that sound the same, but have alternative spellings, which is sometimes the case for person names. It is well suited to disambiguation after specimen labels are transcribed, but before they are imported into a collection management system. There are several manuals available on how to use OpenRefine, but Hill (2016) and Sterner (2019) were specifically written for people working on collections.

## Best practices

**Figure 4.**
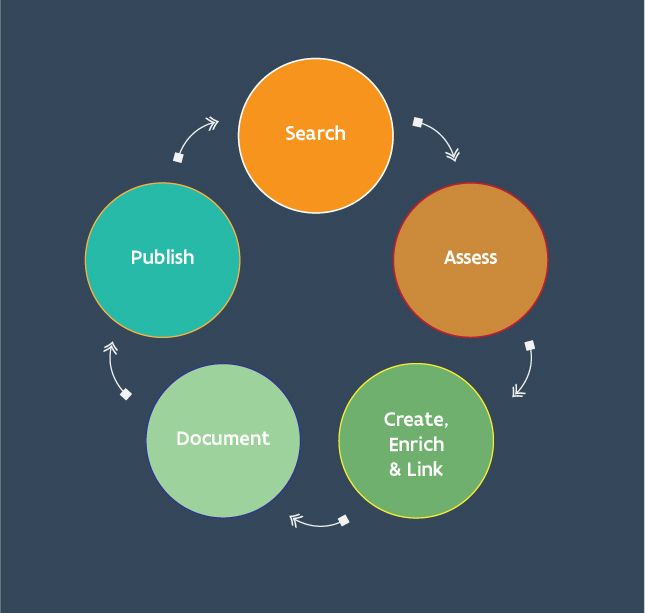
Disambiguation is a cycle. Enrichment of the data feeds off itself leading to further disambiguation. As more names are disambiguated and more biographical data are accumulated, it becomes easier to disambiguate more names.

The disambiguation process can be visualised as a cycle of disambiguation that enriches and links data through identifiers with the aim of completing a roundtrip of data improvement (Fig. 4). The entry point into this cycle may vary depending on the trigger for the disambiguation event and some disambiguation work may also be more focused and, therefore, restricted to one section of the cycle.

The process of disambiguation is inherent to most aspects of working with people names including data capture, management and analysis. For example, the moment a person's name is entered into a collection management system, a decision is made about how that name is recorded and if it should be associated with other names and records in the system or with external resources. Whether entering data for newly-collected specimens or bulk enhancement of historical specimens already held in collections, ensuring that the people's names are unambiguous and, where possible, associated with an identifier, is an important aspect of collection data management.

The process of disambiguation for natural history collection data may be triggered by a wide range of activities undertaken by many individuals including curators, data managers, researchers and citizen scientists. The range of triggers and the kind of activity being undertaken may result in people entering the disambiguation process at various points, carrying out work within all or part of the disambiguation process, with each part of the process being more or less iterative.

In this section, we provide an overview of the steps that may be involved in disambiguating people's names and the individuals to whom they refer, namely: preparation, prioritisation, searching, assessing, creating, enhancing, linking, documenting and publishing. Recommendations for the disambiguation process have been identified and are included within the relevant stages of disambiguation below. In addition, recommendations have been identified for data capture and management; implementing these recommendations will reduce the need for disambiguation in the future.

### Before you start

The need for disambiguation could be triggered by any number of activities, events or research needs. For example,

- an institution may decide to systematically disambiguate their collection, perhaps to improve the data quality and findability of their specimens;
- disambiguation might be the focus of a specific project, such as evaluating the contribution of women to taxonomy or studying the history of scientific exploration of a region; or
- disambiguation might be caused by a specific event, such as the death of a collector, the release of data from another collection or a novel data analysis.

#### Preparation

Depending on the activity and the trigger for disambiguation, it may be helpful to consider some preparatory steps to aid the process. Creating batches or clusters of records that require disambiguation will make the process easier, faster and more accurate. Clustering might be on date, collecting location, the taxonomy of the specimens, co-collectors or anything that will enrich the batches for one or a few people. These aggregated records will often provide additional information and context, as well as providing ranges and variation within the data. Additional disambiguation techniques may also use batches of digital specimen images that are processed through image analysis software (e.g. trained machine-learning models) to extract data specifically for disambiguating the names within the specimen records (Walton et al. 2020).

**Recommendations**

- Batch or cluster records/images to increase the information and context of each record and to reduce the likelihood of repeating the disambiguation process for related records.

#### Prioritisation

Determining the most appropriate order to disambiguate will save time, maximise utility of the data and reduce the overall effort. Disambiguation work will be most efficient if it is focused on a particular taxon or geographic area. Working on subsets of clustered data provides clear boundaries around the people involved and their co-collectors, identifiers and publications. If a more general disambiguation is envisaged, then prioritising the most frequently occurring names means a large proportion of specimens will be resolved quickly. On average, if you can disambiguate 3% of the most prolific collectors or identifiers, this will connect those people to 80% of specimens in a collection (Groom et al. 2020).

In some cases, a person's name is relatively distinctive, at least within a collection. Names such as these may be possible to link to an identifier directly through automatic string comparison. Additionally, as disambiguation supports further disambiguation, roundtripping of identifiers into collection management systems and other databases helps to simplify and accelerate further disambiguation.

There is also a good case to prioritise the disambiguation of names for which undocumented knowledge is available, such as for people who are alive, recently died or for whom an oral history still survives.

It should be remembered, however, that due to historic data practices, many of the participants of biological collections have been under-reported. Collection should not reinforce this discrimination by always focusing on the most conspicuous people, even if this represents the easiest path.

**Recommendations**

- Prioritise disambiguation activities that have high value for research and that balance representation of collectors.
- Integrate disambiguation processes, activities or outcomes within new or existing transcription activities to maximise efficiencies.

### Search

An element of searching is part of most disambiguation processes. Indeed, a search could be the trigger for disambiguation when a name cannot be resolved by the resulting data discovered during the search.

#### Searching in collection management systems

Undertaking a search in an institutional collection management system is often the first step in the disambiguation process (Fig. 5, also available in a PDF format Suppl. materials 1, 2). The data structure in a collection management system will often have an impact on how an initial search is undertaken and it may be necessary to carry out a search in several tables and fields to ensure that all relevant records and information are found. However, where an individual is recorded in a system under more than one role, such as collector, identifier or staff member, it might be easiest to disambiguate the application of the name by one role at a time. For example, if the name is linked to both the collecting and determining of specimens, query for occurrences of the name as collector and disambiguate those first.

To differentiate between two or more people with the same name, it is useful to sort the occurrence records by different values, such as collecting date, collecting numbers and locality (and, in some cases, co-collectors and taxa). To ensure the results of the search include this information, it may be necessary to define the structure and content of the results within the search itself. Therefore, when searching a collection management system:

- Consider which data are expected in the results and how they will be exported.
- Look for patterns in collecting dates, collecting numbers and/or localities that identify the activities of an individual. Depending on the number of records, it may be helpful to chart collecting dates or map collecting localities to reveal patterns in collecting activity.
- Group records that appear likely to be collected by the same individual by annotating each row or splitting the data into separate datasets. The ability to easily view the range of specimens already collected by an individual to determine whether they were all collected by the same individual or to match a new specimen to the correct collector record in the collection management system will be important for the efficiency and accuracy of any disambiguation process. It will often be necessary to find more information than is available in a system to disambiguate names.
- Try to establish the identities of the different individuals by searching online or in other resources.
- Be mindful that apparent anomalies in collecting localities can sometimes be explained by travel for personal or professional reasons. Knowledge of where a collector's extended family resided can provide a justification for travel to a given locality, especially for non-professional collectors in the 19^th^ century or earlier.

**Recommendation**

- Develop a good strategy for searching, with an understanding of the methodology, limitations and context of each collection management system used.

#### Searching online

Once a target for disambiguation has been identified and any local information, such as related specimens, has been collated, the easiest option is to use a search engine, such as Google and search for the person’s name. For notable people, this may be all that is required to establish who they are and link them to an existing identifier. However, such searches often need to be made more specific by the addition of biographical material, such as the name of an institution where they worked, their birth or death year or the name of someone they worked with. Such searches will often lead to additional information that can be used to refine searches and/or query databases, such as the Biodiversity Heritage Library, the Internet Archive, FamilySearch, Ancestry.com, bibliographic databases, Wikipedia and Wikidata (see the Sources of Information section below).

Each of the resources mentioned above differs in its scope and limitations. A person may have an extensive record in one dataset, but be missing entirely in another. Or, for example, Google Scholar might retrieve very different results to Biodiversity Heritage Library full text search. Search results from different datasets will often complement each other and, as biographical and specimen data are continually growing in availability on the internet, searches can always be revisited with renewed chances of success.

**Recommendations**

- Develop an online search strategy with documented methodology that is sensitive to the limitations and context of each online resource used.
- Become familiar with candidate search resources and use them in concert to refine your evidence.

### Assess

The assessment of a manual disambiguation process is based on the experience of the person carrying out the disambiguation. Experience only comes with practice and learning and even very knowledgeable disambiguators can be misled by the data. It is important to judge whether, on the balance of probability, the existing data on both the specimen and the person are sufficient to make the link between these two entities.

The criteria used might include dates, places, taxa, handwriting, label format and co-collectors, but the weight placed on each data type is up to the disambiguator and their knowledge of the specimen and the person they want to link to it. Disambiguators need to be self-critical and be willing to re-evaluate and return to decisions in the light of new information. It is important to document the data as they are uncovered, but it may not be possible to document the complete trail of breadcrumbs that often precedes the discovery of a person and their biography (Fig. 5, Suppl. materials 1, 2).

**Recommendation**

- Be prepared to accept failure. Disambiguation is an iterative, cumulative and re-affirmative process, but it is not absolute.

### Create, enhance and link

When disambiguating the names of people attached to specimens, it may be necessary to create new records in a collection management system. It may also be possible to enhance existing records, based on new information gained during the disambiguation process. When creating a record in a collection management system, there may be specific protocols and constraints on how the data should be entered. The use of verbatim fields can provide useful information on how a person's name has been written on specimen labels that can aid future disambiguation. Verbatim fields can also be used to record names that cannot be unambiguously assigned to a single individual.

Where possible, enhance the record for an individual with information that will aid the correct use of the record in future. If you have specimens that have been collected by the individual, then it is usually possible to provide dates which bound the period of activity of that person (i.e. floruit dates, sometimes abbreviated to fl.). Likewise, the geographical region and taxonomic interests of a person can be determined and added to the biographical record. The disambiguation process will also often require new identifier records to be created in the authority resources. When creating or enhancing a record in Wikidata, aim to include at least one reference. If you are creating a Wikidata record for someone for whom you have very little information, then the reference could cite a specimen collected by them.

Linking the local record in a collection management system with the identifier record in the authority resource is key to embedding the disambiguation into the data. The process of creating the link may vary depending on the collection management system and the resource. If there is a central table of people records within the system, this may be the most appropriate place to hold the Wikidata or ORCID identifier (the link to the global resource). The structure and functionality provided by the link may also impact the level of information stored locally in the system. Some locally maintained data may be the preferred option if the local system is quite isolated or there are sensitive data involved.

**Recommendations**

- Capture the person's name verbatim from the label in the specimen record and treat disambiguation as a secondary enrichment process.
- Link to and contribute to large communal biographical resources, such as Wikidata, rather than large local stores of biographical information.
- If an identifier does not exist for a person, create one, ideally a Wikidata Q Identifier with a minimum of one reference.
- If an identifier already exists, check whether any information gained from the disambiguation process can enhance the authority identifier record.

### Document

If a disambiguation decision has not been thoroughly documented, explained and referenced, there is a risk it may be undone in the future. It should be considered how the decision should be recorded. That is not to say that all the information needs to be documented in the same place. Enriching a collection management system is suitable for specimen related annotations; however, biographical information may be better suited to be documented in Wikidata or even Wikipedia, if the person is notable enough.

The person who made the assessment and the date should be recorded; in most systems, this is automatic. Notes, flags and tags all might be useful to document levels and sources of uncertainty. If disambiguation attempts fail, it is equally important to record the reason and information discovered in the process. To indicate the reasons for a failed disambiguation, the following flags might be used:

- No biographical details are available for collector's/determiner's name.
- The specimen has insufficient information to attribute to a collector/determiner.
- There are candidate people, but it is suspected that there is an error on the specimen label or in the biography of potential collectors/determiners that prevent disambiguation.

Sometimes, names written on specimens cannot be disambiguated. For example, it can be difficult to separate a husband and wife who often travelled together and have the same initials. In some collection management systems, it might be possible to create a collecting team, but it is not appropriate to record such a team in Wikidata. It is not uncommon to fail at disambiguation, but to have enough information to limit the choice to a small number of people. The results should then only be recorded at the specimen level, although information relating to the potential confusion of individuals could be documented in the person records of a collection management system.

**Recommendation**

- Record the results of the disambiguation whether or not success was achieved.
- Enrich public domain resources, such as Wikidata, because this benefits global disambiguation activities.

### Publish

Institutional collection management systems contain a wealth of carefully-curated data researched by people with extensive specialist knowledge. This may include years of work ensuring that the person names are unambiguous. If this work is not published, this information will not be made available to the wider community. Publication processes in the past have often made it difficult to include this information, particularly when submitting data to aggregators.

However, the addition of the Darwin Core fields “recordedByID” and “identifiedByID” mean that the identifier for the collector and the determiner can (and should) be included with published data. These terms are also available for use by institutions, enabling the inclusion of person identifiers in institutional portals. These identifiers can, thus, be included in data downloads and on printed specimen labels (Fig. 6).

**Recommendations**

- When publishing a person's name, include the full name and any titles and suffixes.
- Publish the identifiers attached to the records when publishing data on portals or to aggregators.
- Where possible, include the person identifier for the collector on the labels for new specimens. This might be done as part of the accessioning process.

## Sources of information

People and their lives can be described through various types of data, which can be obtained and cross-referenced from multiple sources. Below is a non-exhaustive list of the different characteristics that may be leveraged for disambiguation. These are sometimes only understandable in the context of the era and culture of the people to whom they refer. The person doing the disambiguation must make a judgement as to which sources are likely to provide fruitful results.

- Depictions: portraits, photographs
- Physical traits: ethnicity, sex, other distinguishing features
- Identifiers: names, aliases, signature, persistent URIs
- Linguistics: language, dialect, vocabulary
- Interests: taxonomic groups, localities
- Awards: prizes, grants, titles
- Positions/roles: jobs, qualifications, memberships
- Birth-death: dates of, active lifespan, attendances
- Proxies: parents, children, siblings, spouses, friends, colleagues, students, supervisors, co-authors, co-collectors
- Material evidence: publications, presentations, specimen labels, letters, field books, certificates, collection registers

Below, we outline the most relevant resources of biographical information for biological collections. There are many more. With experience, disambiguators will discover suitable sources for the category/ies of people they frequently work on. The lengths one is prepared to go to disambiguate someone depends on the nature of the project, the importance of the person and the likelihood of success.

### Genealogical websites

The genealogical community and their research are an extremely helpful resource for disambiguating people. The decades of work by this community have culminated in multiple websites that contain a wealth of interconnected data on people, including family relations, birth, death and life event dates, as well as links to primary source documentation that support those data. Examples of these websites include Ancestry.com, Billiongraves.com, Familysearch.org, Findagrave.com, Findmypast.com, Geni.com, Myheritage.com and Wikitree.com to name but a few. The data and linking contained in these websites can greatly assist with the disambiguation of people associated with natural history specimens.

### Miscellaneous publications, catalogues and field notes

Co-collectors are often co-authors of scientific publications. A single collector's surname can be difficult to disambiguate, but a pair of surnames is often unique. Resources such as Google Scholar facilitate searches across scholarly publications for pairs or teams of authors. Once potential matches have been found, the publications themselves often reveal more information about the collectors, including their full names and institutional affiliations. The field notes and diaries of some collectors may also be publicly accessible online. *Indices Collectorum* are another source of data. These are a published catalogue of a collector or several collectors' activity. They may be associated with *exsiccata* that may have once been offered for sale (Triebel and Scholz 2022). The Biodiversity Heritage Library (see below) is an excellent source of these. Such resources often contain additional information about expeditions and the collectors themselves. These notes also link specimens together, potentially making them suitable for bulk disambiguation of numerous specimens.

### Biodiversity Heritage Library (BHL)

BHL is the world's largest online repository of biodiversity literature and archival materials. It is a global consortium of over 500 libraries and publishers, who have together made over 60 million pages freely accessible online. Users can search the contents of the Library in two ways: by searching the catalogue for publications (and filtering by author, date or subject) or via full-text search, which searches the OCR text across all 60 million pages. This opens up information on little known people and provides valuable biographic references useful for disambiguation. The knowledge gained can add to the totality of evidence and improve the confidence in the disambiguation. BHL also shares its contents with the Internet Archive, which provides yet more content that might have relevance to disambiguation.

### Taxonomic Literature II (TL-2)

TL-2 is a guide to the literature of systematic botany published between 1753 and 1940 and was originally a print series in fifteen volumes published by the International Association for Plant Taxonomy (IAPT). A digital version of TL-2 has been made available online by the Smithsonian Institution Libraries. It contains detailed biographies of taxonomists, including their publications, their employment history and the institutions where their specimens have been deposited. It is an invaluable source of information about people publishing in a specific field within a specific time period.

### Specimens

Many institutional collection databases have an online portal and some have associated biographical information. The Global Biodiversity Information Facility (GBIF) aggregates biodiversity data from institutions across the globe, thus making it accessible and discoverable within a single portal. The digital occurrence data records, inferred from specimens and other material samples available on GBIF, are limited by the extent of documentation by the data provider for the collection. However, images of specimens from these host collections can be a useful resource in their own right. The specimen labels captured in collection images can contain critical information that has not been transcribed or included in collection data, such as "Mrs" or Jr" (see Prejudices & Biases section above). Specimen labels can also be used to verify spellings of transcribed names and other data.

### Wikipedia

Wikipedia is an openly-licensed online encyclopaedia to which anyone may contribute. Wikipedia content is also indexed and ranked highly by search engines. This ensures that, if an article on a collector exists, that article will be one of the first returned search results. Wikipedia has more than three hundred language versions, with the English language version being the largest. These different language versions have independent content, so information missing in one language version may be present in another. Helpfully, Wikidata provides a bridge between the different language versions. Wikipedia collates knowledge about people in an accessible, editable, online resource and is, therefore, of great assistance in disambiguation efforts. As Wikipedia is a centralised resource, any contributions made to Wikipedia are more visible and are likely to have more impact than contributions made to more specialised platforms. Nevertheless, not all people can be included within Wikipedia due to the encyclopaedia's notability criteria. In Wikipedia, people are presumed notable if they have received significant coverage in multiple published secondary sources that are reliable, intellectually independent of each other and independent of the subject. Many contributors to natural history collections fail to meet these criteria, even if they meet the criteria for Wikidata (see Wikidata section above).

### Virtual International Authority File (VIAF)

VIAF is a large authority file created from a consortium of international libraries who contribute their own local authority files. VIAF consolidates authorities from its sources and, where possible, aggregates them under a single VIAF ID related to a single person. However, where biographies have not been linked, there may be multiple VIAF IDs for a single person. VIAF is run by OCLC (the Online Computer Library Center), a non-profit membership organisation. As Fig. 2 demonstrates, VIAF plays an important role in the landscape of identifiers, but it is far from a universal identifier for biologists.

### International Standard Name Identifier (ISNI)

ISNI is a person identifier for anyone involved in the production of creative works. This includes authors, artists, musicians and their producers and publishers. ISNI is also an ISO Standard Identifier (International Organization for Standardization 2017). This means it has a larger scope than VIAF, but in the context of biological collections, the people included are mainly the authors of publications on science and natural history.

### Other identifiers

Other identifiers useful to collection disambiguation work include those in databases, such as Harvard Index of Botanists, International Plant Names Index (IPNI), ZooBank, Biodiversity Heritage Library and Wikispecies. These have an advantage over those in Wikidata, ORCID, VIAF or ISNI in that the person records present in them are more likely to be linked to specimens in a collection. Most of these other identifiers are also available as Wikidata properties and, therefore, Wikidata can also be used to reconcile identifiers between these databases (Fig. 2).

## Case studies

Here, we describe some specific disambiguation projects conducted by the authors. These case studies illustrate some of the problems, processes, sources of information and benefits of disambiguation.

### Collaborative disambiguation of North American bats

In a project to enhance currently published data for the rhinolophid and hipposiderid bats, some authors of this paper held a workshop with bat researchers and collection managers on 1 December 2020 (Mast et al. 2021). Our goal was to verify the identity of historic and present-day people who collected or identified specimens of these taxa. During this disambiguation marathon, workshop participants uncovered problems, solved them in a virtual group setting and came to appreciate the value of this exercise. Below, we outline some of their discoveries.

Using Bionomia to explore specimens of bats labelled with the seemingly unique string "*Geoffroy Saint-Hilaire*", workshop participants were able to discover patterns and outliers in collection and determination dates which indicated that they were not all collected or determined by one individual. By examining biographical data in Wikidata for Geoffroy Saint-Hilaire, participants discovered that there were, in fact, three people who shared at least parts of the same name: the father Étienne Geoffroy Saint-Hilaire (1772–1844), the son, Isidore Geoffroy Saint-Hilaire (1805–1861) and the grandson Albert Geoffroy Saint-Hilaire (1835–1919) who all collected bats. By cross-referencing birth and death dates, participants were able to decipher which bat specimens were most likely collected by which of these three family members. These deductions later served to help inform a second team of participants who were charged with verifying georeferenced collection localities.

Linking people to specimen records in a publicly accessible, web-enabled environment like Bionomia (and Wikidata) resulted in surreptitious discoveries. Harry Hoogstraal (1917–1986) was an American zoologist and prolific collector of specimens, particularly in the tropics. Prior to the workshop, many of his rhinolophid and hipposiderid bat records had already been linked to him via Bionomia through his Wikidata Q Identifier, Q5669784. As a result of search engines indexing this content, the string “Ibrahim Helmy” was made discoverable on the Internet, plainly seen as a co-collector of Harry Hoogstraal. This was a necessary clue that led to the discovery that Ibrahim Helmy co-authored, “The Contemporary Land Mammals of of Egypt (Including Sinai)”, with the late Dale James Osborn, a Research Associate with the American Museum of Natural History (Osborn and Helmy 1980). Ibrahim led field expeditions in support of the US Navy’s Naval Medical Research Unit Three (NAMRU-3) Medical Zoology programme then stationed in Cairo, Egypt.

**Figure 7.**
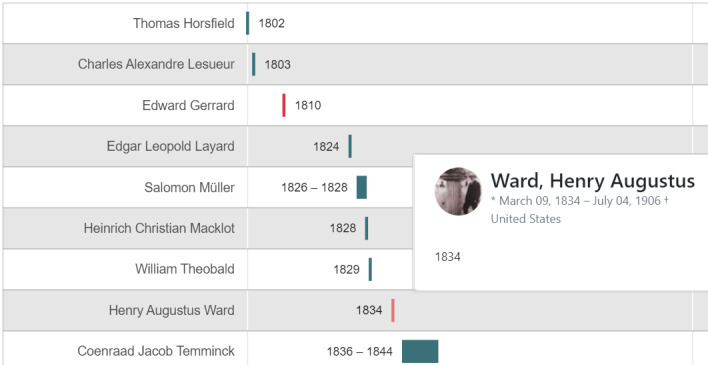
Timeline for the earliest hipposiderid bats (Old World leaf-nosed bats) linked to collectors via Bionomia. The narrow, red bars with the years 1810 and 1834 indicate a problem with these attributed records requiring further investigation. Edward Gerrard was born in 1832 and Henry Augustus Ward was born 9 March 1834.

Workshop participants were able to assign ORCID identifiers and/or Wikidata Q Identifier to over 500 people involved in collecting bats. The breadth of these activities revealed unlikely collection dates for some of the earliest-known bat specimens (Fig. 7), which are now under investigation by collection managers. Cross-referencing readily-, openly- and computationally-available birth and death dates of collectors against dates of collection demonstrates the value of involving the research community in this disambiguation activity.

### The Schimpers

The Schimper family of Baden, now part of Germany, produced four important scientists of the 19^th^ century: the brothers Karl Friedrich Schimper (1803–1867) (Fig. 8a) and Georg Wilhelm Heinrich Schimper (1804–1878; GWHS), their cousin Wilhelm Philipp Schimper (1808–1880; WPS) (Fig. 8b) and his son Andreas Franz Wilhelm Schimper (1856–1901; AFWS) (Fig. 8c). Three of them were of the same generation and all made a considerable contribution to our botanical knowledge. The complexity of disambiguating these names when digitising label data from specimens collected by the Schimpers has been outlined in Bräuchler et al. (2021) and is summarised here.

All four Schimpers collected plant specimens that are now in collections around the globe; indeed, many taxa are named after them. Consequently, their names are frequently mentioned in literature and on herbarium specimens. In many herbaria, only the family name is recorded on the labels, so it is not clear which individual collected the specimen. The presence of an initial on the specimen label does little to identify the individual, given that, despite their different forenames, GWHS, WPS and AFWS all went by the name Wilhelm. The resulting confusion has led to two entries for “W. Schimper” in Harvard University Herbaria Index of Botanists (ID 0094171, 0094172). GWHS and WPS are most likely to be conflated, due to their overlapping periods of activity and the large volume of herbarium specimens either collected, identified or distributed by them: both distributed exsiccatae of their collections, either as gifts, exchange or for sale.

Given the wealth of literature and online information on the Schimpers and the sometimes inconsistent information contained therein, it can be time-consuming to disentangle the information needed to disambiguate these collectors.

The collecting locality provides the best starting point for disambiguation, with GWHS alone having collected in Algeria, Greece, Egypt, Saudi Arabia and Ethiopia. Although the country of collection can be used to determine the collector for a large proportion of Schimper specimens, it does not help with specimens from France and Germany, where all four Schimpers collected. For these, a thorough knowledge of the biographies and collecting activities is necessary for disambiguation.

Knowledge of each collector's taxonomic speciality is also useful but, again, there is some overlap: WPS identified and described the mosses collected by GWHS and even distributed part of his Ethiopian specimens via his exchange society, so WPS' name may also be associated with GWHS' specimens.

Again it is clear that high quality biographical data is necessary for disambiguation, but also that data from the specimens themselves contribute biographical information, so that disambiguation benefits from the process of disambiguation itself.

### Ethel Winifred Bennett Chase

**Figure 9.**
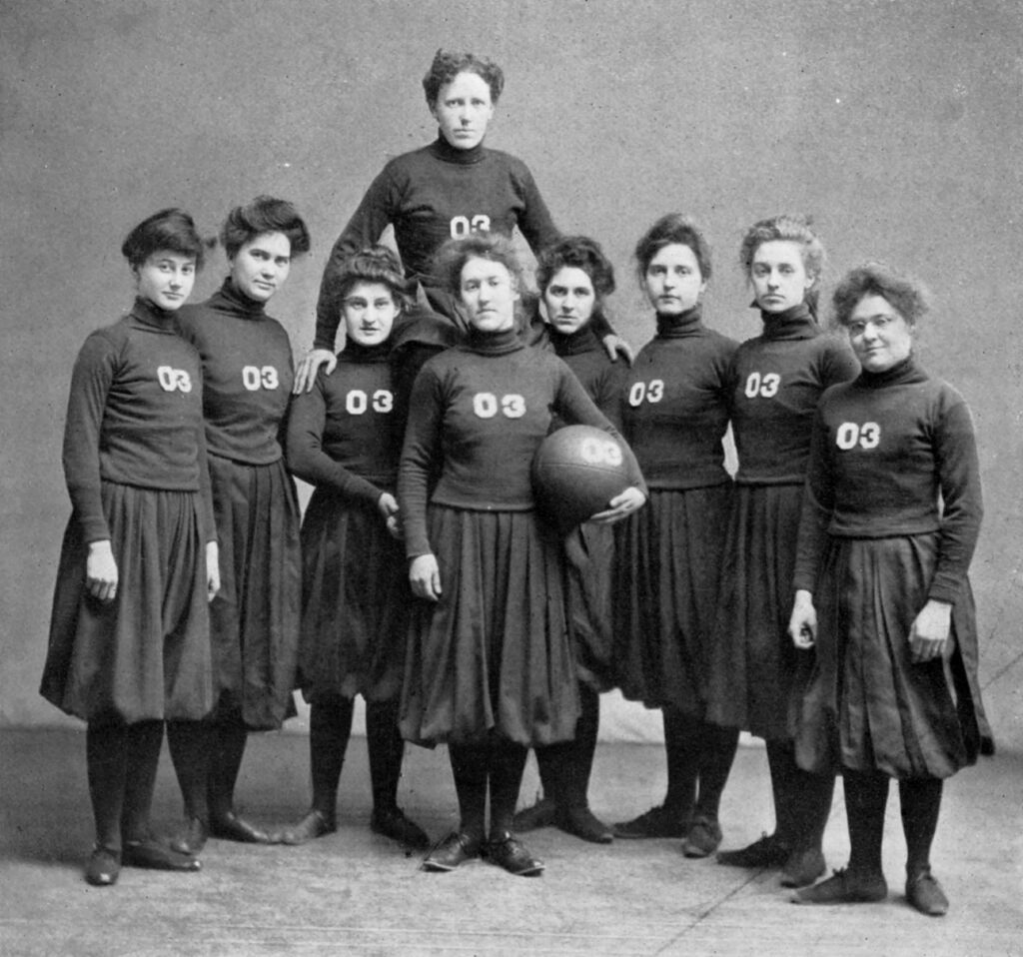
The University of Michigan Class of 1903 Women's Basketball Team with Ethel Winifred Chase, third from the left. It is rare to find pictures of notable women who collected specimens; if they do exist, they are rarely in the portrait style of eminent male collectors. This is the only picture we know of depicting Chase. From the University of Michigan, public domain, via Wikimedia Commons.

Ethel Winifred Bennett Chase was an American botanist, a professor of botany and the Dean of Women at Wayne State University in the United States (Fig. 9). Much of her scientifically significant botanical collecting was undertaken on an expedition to the South Pacific, accompanying the algologist Josephine E. Tilden. However, correctly attributing specimens to Chase can be challenging as she preferred to use her middle name, Winifred. The numerous resulting name strings used by herbaria databases have also led to confusion.

In order to assist with disambiguation, a Wikidata item was created for Chase. This item was linked to the item for Josephine E. Tilden through a statement that the two botanists were co-collectors. This ensures that the Wikidata notability criteria was satisfied for both people. Having a Wikidata item allows Chase's multiple aliases to be listed. It also enables the collation of biographical data, institutional identifiers, databases, websites and scholarly articles as supporting references for statements added to that item.

Various resources were used to research Chase. They include the Harvard Index of Botanists which contained two entries, a JSTOR Global Plants person database entry, the genealogical research website FamilySearch, which provided her exact birth and death date and a full text search of the Biodiversity Heritage Library corpus, which led to the discovery of a scholarly article on Chase (Jones 1966) . This article outlined the existence of historically significant lantern slides made by Chase during the South Pacific expedition. An email to the University of Michigan Herbarium brought these slides to the attention of curators. The institutional knowledge about these slides had lapsed, but as a result of the disambiguation research being shared with the University of Michigan herbarium staff, historical context as well as attribution data were added to the lantern slide collection. A Wikipedia article on Chase was also created collating the knowledge gained during the disambiguation process.

### Dr. Dorothy Swales

Dr. Dorothy Swales was a Canadian botanist and the first female curator of the Macdonald College Herbarium (now known as the McGill University Herbarium). Born in Quebec in 1901, Dr. Swales would attend Macdonald College (later part of McGill University) to earn both undergraduate and graduate degrees in Plant Pathology and Bacteriology, respectively. She would later earn her PhD in Mycology from the University of Manitoba. During her tenure, from 1964 to 1971, Dr. Swales collected extensively throughout Quebec and the Northwest Territories with a specific focus on plants found in the Arctic and sub-Arctic regions (e.g. Fig. 10). Under Dr. Swales, the Herbarium's collection expanded extensively and was enriched through international exchanges of botanical specimens from herbaria in the U.S.S.R., Sweden and Denmark. After retiring in 1971, Dr. Swales continued to engage with the botanical science community at McGill University as emeritus curator.

Despite Dr. Swales' contributions to the field, both as a botanist and herbarium curator, the lack of a significant or unified digital presence made piecing together the story of her botanical collections and collaborations difficult. Her correspondence, notes and specimens are currently housed at the McGill University Herbarium, but many items in the collection are yet to be digitised. Both professionally and across botanical specimen sheets, Dr. Swales was listed using different variations of her name such as Dorothy E. Swales, Swales Dorothy E, D.E. Swales, Mrs. W.E. Swales (her husband was Dr. William Swales) and Dorothy Newton (her maiden name). Although a search using a variation of her name in individual databases, such as GBIF or Canadensys, might return a result of digitised specimens attributed to Dr. Swales, the different versions of her names, as well as the absence of links between her collections housed in different institutions and available across digital platforms, creates a problem for telling a fuller story of her career as curator and botanist.

The disambiguation process first required the creation of a Wikidata profile and Q number and then the creation of a digital profile on Bionomia. Dr. Swales has now been unambiguously linked to specimens (either as collector or determiner) across 15 organisations. This work has collated her contributions to the McGill Herbarium during her tenure and drawn her collections under one Bionomia profile. Further information was found from her obituary, Google Scholar and resources on McGill University's history relating to the Herbarium and Macdonald College. Unified attribution for Dr. Swales enables a more detailed and clearer narrative of who she was as a botanist, curator and educator. Broadly speaking, as more archival documents (e.g. curator correspondence, field notes) are digitised, the solid establishment of a digital presence will make it easier to add supplemental material information about botanists and their collections, thereby enriching the information to be used for researchers. This will be especially helpful for those focused on the history of women in botanical science.

## The next steps for collections

The effort to disambiguate people's names should decrease over time. In fact, it is part of the evolution of collection data management that ends when people are identified as unambiguously as possible. Full disambiguation is many years off, but rapid progress can be made for the vast majority of cases as outlined by Groom et al. (2020) and Güntsch et al. (2021). Below, we propose a number of specific objectives for the next phase of activity. These objectives, which are presented using the SMART framework (Specific, Measurable, Achievable, Relevant, Timely), are written as a direct challenge to collections and their funders to motivate this critical work.

### Objective 1: Promote the use of person identifiers in local, national or international outreach, publishing and research activities

This is measurable through the number of person identifiers used in publications and other research outputs. The objective is achievable because the potential for using disambiguated person data (particularly historic data) in scientometrics and biodiversity informatics has not yet been fully realised. This is timely because aggregated person data help us to answer new questions about the relevance of collections, their scientific output and their sociopolitical histories, in addition to supporting policy.

### Objective 2: Increase the number of collection management systems that use person identifiers

Modifying data management systems to accommodate person identifiers is relatively simple for most systems, though more sophisticated use of indicators for matching, merging and comparing data is more demanding. Improving software systems is achievable and measurable because software systems are constantly evolving and new ones emerge regularly. Building-in person disambiguation functionality at the design stage is the best strategy. It is timely because collections are increasingly requiring more clarity on person data and software is needed to close the roundtripping cycle.

### Objective 3: Increase the number of living collectors registered and using an ORCID identifier when contributing to collections

The uptake of ORCID identifiers can be measured internally by institutions, but also by their use in publications linked to collections and in Wikidata and GBIF. This can be achieved through institutional policies, such as an acquisition policy or data management plan and through promotion of ORCID to collectors who may not currently appreciate how it benefits them. It is relevant because institutions are increasingly being compelled to better manage issues, such as data protection, data sharing and benefit sharing. The advent of GDPR has raised awareness of our rights and responsibilities regarding data on people; increasing the use of ORCID identifiers provides a timely mechanism for better managing person data in-line with GDPR.

### Objective 4: Undertake disambiguation in the national languages of many countries

This can be measured by the number of languages for which software, data and training materials are available and can be achieved because Wikidata and Bionomia are already multilingual systems. It is relevant because the disambiguation of names is particularly important for people whose languages are not in Latin scripts and because providing disambiguation guidelines and resources in languages other than English would significantly support adoption. It is also timely because, in the spirit of the Convention on Biological Diversity, the institutions in the Global North have a responsibility to support those countries in the Global South from where many specimens have been obtained.

### Objective 5: Increase the number of identified people on Wikidata linked to collections

This is measurable through counts of people and their links on Wikidata, particularly those identified as biologists. It can be achieved through training, community events and projects dedicated to using the results. It is relevant and timely because collections acknowledge their responsibility to recognise the diversity of people who contribute to them and because the tools, specimens and biographical resources are increasingly available digitally online.

### Objective 6: Increase the number of people in collections with expertise in person disambiguation

This is measurable through the number of people attending training events on disambiguation of collections and the amount of disambiguation being done. It can be achieved through in-person and online training events, particularly coupled to collections management and informatics conferences, such as those of the Society for the Preservation of Natural History Collections (SPNHC) and the Biodiversity Information Standards (TDWG) organisation. It is relevant because it will enable collections staff to better manage biographical data in their collection and it is timely because it is increasingly easy to disambiguate people and a concerted effort between collections will help the whole collections community.

### Objective 7: Collaborate towards an exchange standard for attribution data

To roundtrip person data effectively, a data exchange standard is needed, together with tools for data managers to facilitate decisions about what to confidently accept and what to reject on the return trip. This exchange standard should include data on the source from which new assertions were derived, when they were made, who made them and, ideally, what corroborating evidence was used. It is achievable thanks to the existing W3C Web Annotation Data Model, a model for nanopublications and the report on attribution written by a joint working group of the Research Data Alliance (RDA) and TDWG organisations, which provide a timely foundation for standards development (Thessen et al. 2019).

## Conclusions

The current informatics landscape for the disambiguation of people makes it possible to imagine a future where the whole of a person's scientific output is connected. The tools and infrastructure exist to enable and democratise disambiguation of people in collections and there is a clear need. Unlike some other areas of biodiversity informatics, person name disambiguation is an action to which all organisations can contribute and on which lasting and impactful progress can be made. As collections are further digitised, disambiguation will continue, motivated by all the benefits outlined above. We recognise that more work is still needed to disseminate the model for how to do this work, how to share and use these data and how to update current standards of practice that include these identifiers from the beginning. The more people who are disambiguated, the easier the process becomes and the more benefits accrue. While it is likely that tools, databases and collections will change, the broad coalition engaged in disambiguation globally means that there is no single point of failure and we see a bright, interlinked future for collections in which the identities of people will play a pivotal role.

## Supplementary Material

D0FC624D-A03D-5792-AADE-D6286121FFF7 10.3897/BDJ.10.e86089.suppl1 Supplementary material 1 A disambiguation strategy Data type flow diagram Brief description A diagrammatic representation of one disambiguation strategy. Strategies vary considerably depending on the name being disambiguated, the dates involved, the taxonomy of the specimen, the collecting locality and the collection it is held in. Abbreviations: Harvard University Herbaria Index of Botanists (HUH); International Plant Names Index (IPNI); Royal Botanic Garden Edinburgh (RBGE). File: oo_730092.pdf https://binary.pensoft.net/file/730092 Elspeth M. Haston, Christian Bräuchler, Robert WN Cubey, Mathias Dillen, Pieter Huybrechts, Nicole Kearney, Niels Klazenga, Siobhan Leachman, Deborah L. Paul, Heather Rogers, Joaquim Santos, David Peter Shorthouse, Alison Vaughan, Sabine von Mering, Quentin Groom

94C4723F-35D9-5CC0-AC76-90DA3FC01F81 10.3897/BDJ.10.e86089.suppl2 Supplementary material 2 Disambiguation flow example Data type flow diagram Brief description A real example of how a name string is disambiguated and the steps taken in documenting it. File: oo_730093.pdf https://binary.pensoft.net/file/730093 Elspeth M. Haston, Christian Bräuchler, Robert WN Cubey, Mathias Dillen, Pieter Huybrechts, Nicole Kearney, Niels Klazenga, Siobhan Leachman, Deborah L. Paul, Heather Rogers, Joaquim Santos, David Peter Shorthouse, Alison Vaughan, Sabine von Mering, Quentin Groom

## Figures and Tables

**Figure 1a.**
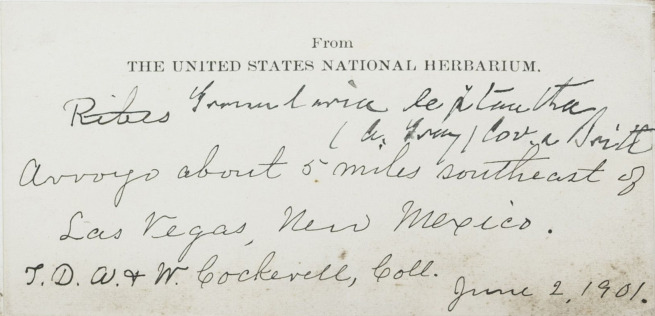
The label of a specimen of *Ribesleptanthum* A.Gray collected by Theodore Dru Alison Cockerell and Wilmatte Porter Cockerell from The New York Botanical Garden Herbarium (NY) where only her husband was documented. URI:http://sweetgum.nybg.org/science/vh/specimen_details.php?irn=4063571. Catalogue Number 3771543 (©The New York Botanical Garden, CC-BY-4.0).

**Figure 1b.**
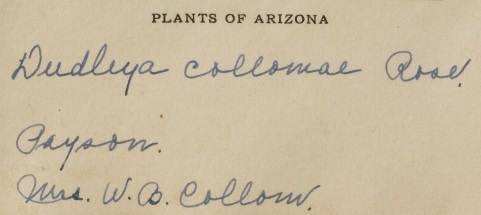
The label of a specimen of *Dudleyacollomiae* Rose collected by Rose Eudora Collom from the Royal Botanic Gardens, Kew where the loss of the prefix “Mrs” has led to the specimen being mis-attributed to her husband W. B. Collom. URI:http://specimens.kew.org/herbarium/K000838434. Catalogue Number K000838434 (©Royal Botanic Gardens, Kew, CC-BY-4.0).

**Figure 1c.**
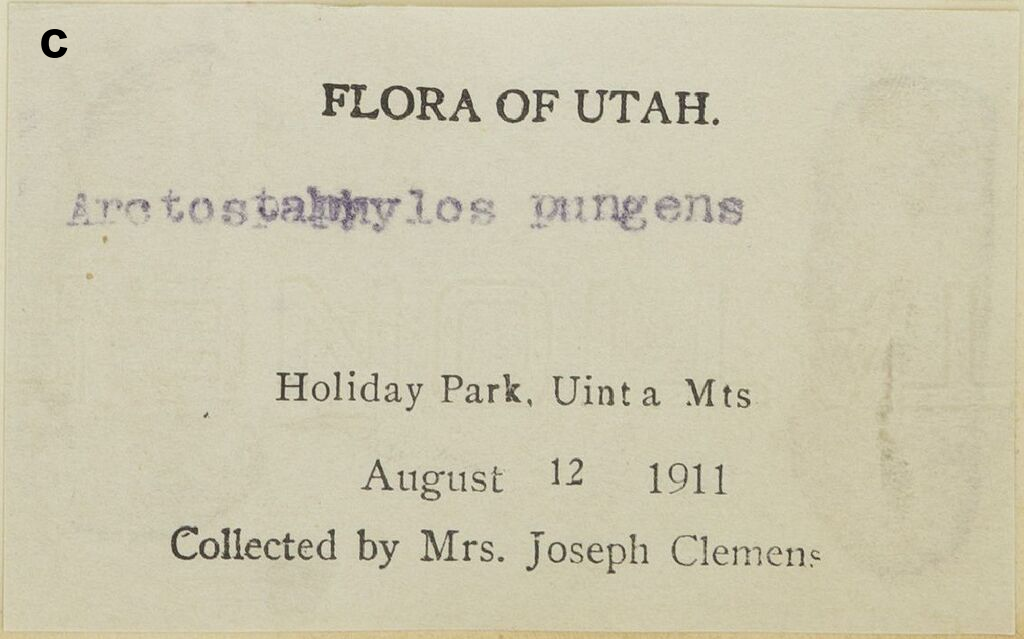
The label of a specimen of *Arctostaphylospatula* Greene collected by Mary Strong Clemens from California Botanic Garden Herbarium, where the loss of the prefix “Mrs” has led to the specimen being mis-attributed to her husband. URI:https://cch2.org/portal/collections/individual/index.php?occid=3895834. Catalogue number RSA0178692 (©California Botanic Garden Herbarium, CC BY-NC-SA).

**Figure 1d.**
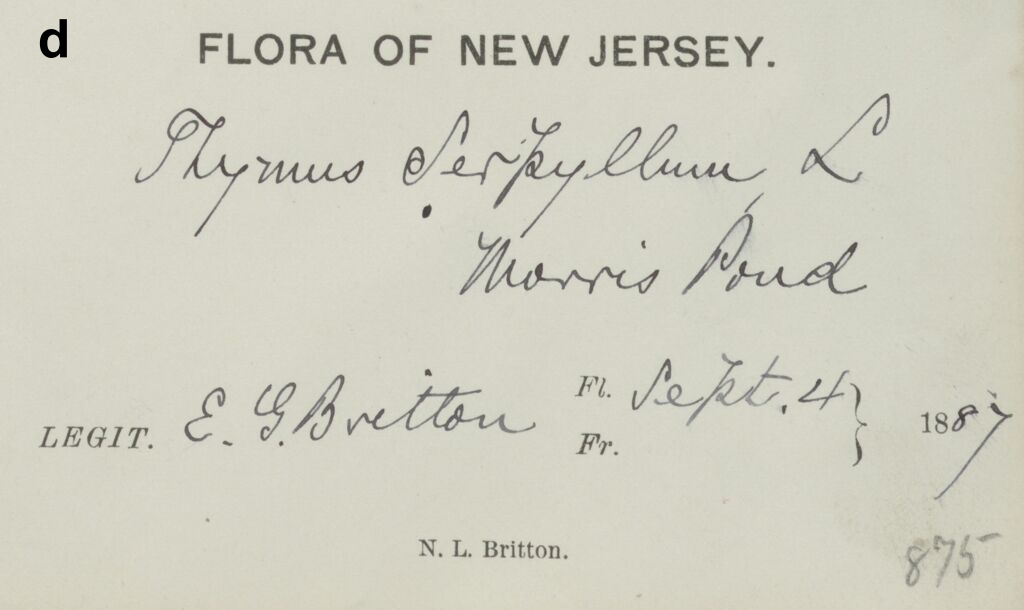
The label of a specimen of *Thymusserpyllum* L. collected by Elizabeth Gertrude Britton in the collection of the National Museum of Natural History, Smithsonian Institution Herbarium, but attributed to her husband Nathaniel Lord Britton in the collection of the National Museum of Natural History, Smithsonian Institution. URI:http://n2t.net/ark:/65665/32616a5e9-2143-490a-a0b3-a5d2546376de. Catalogue number US 132144Z (©Smithsonian National Museum of Natural History, CC-0).

**Figure 5a.**
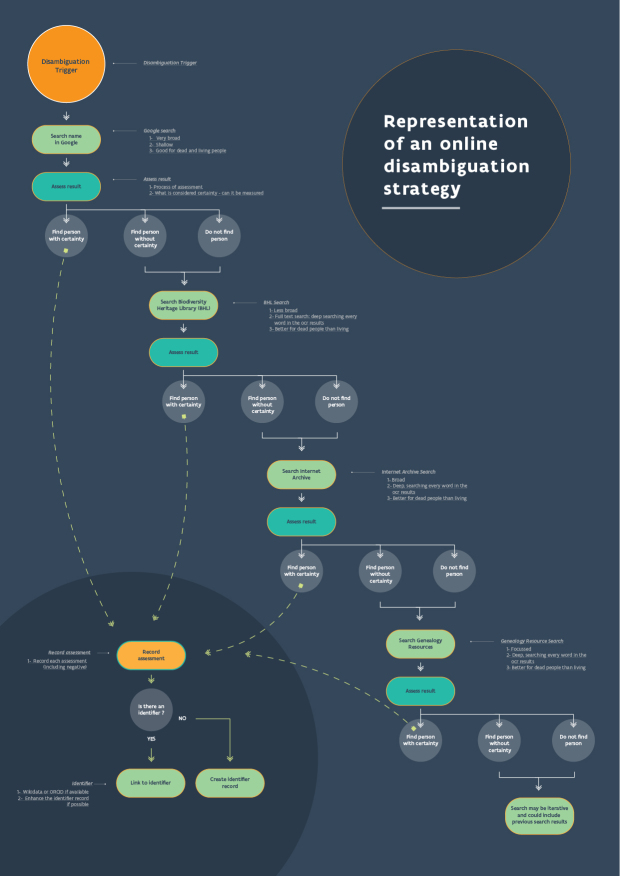
A diagrammatic representation of one disambiguation strategy. Strategies vary considerably depending on the name being disambiguated, the dates involved, the taxonomy of the specimen, the collecting locality and the collection it is held in.

**Figure 5b.**
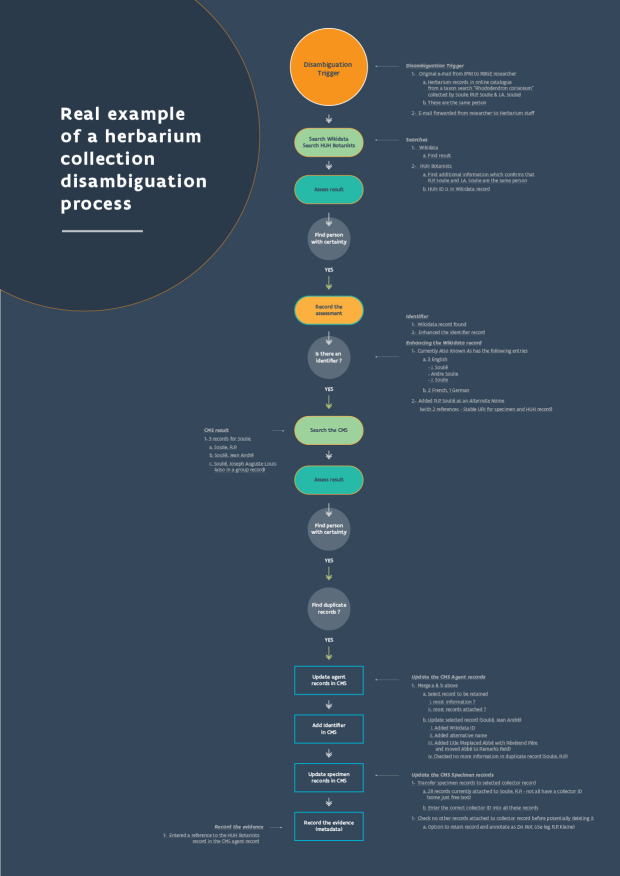
A real example of how a name string is disambiguated and the steps taken in documenting it.

**Figure 6a.**
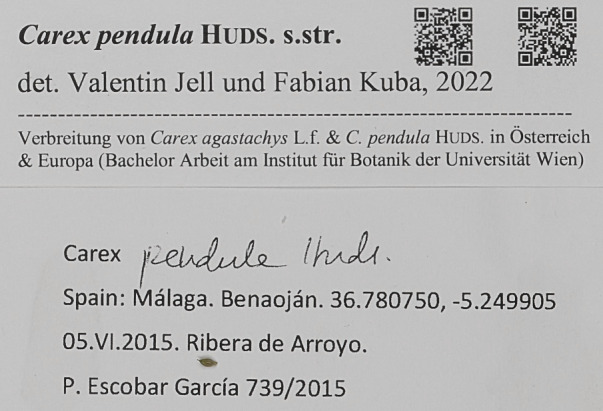
From specimen https://w.jacq.org/W0132025 (©Natural History Museum, Vienna, CC-BY 4.0).

**Figure 6b.**
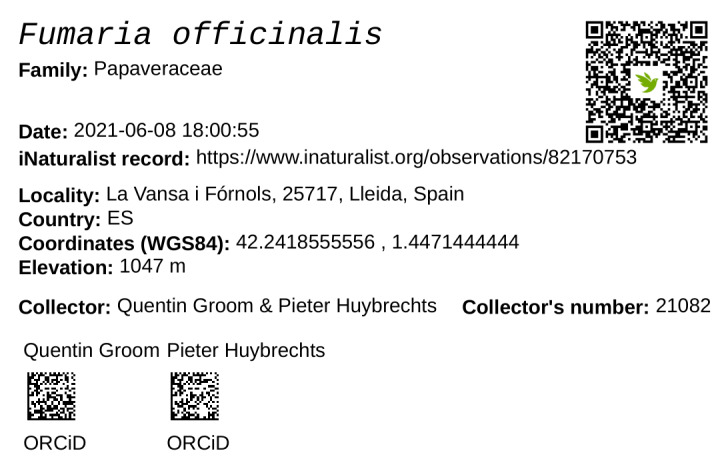
From Meise Botanic Garden (CC-0).

**Figure 8a.**
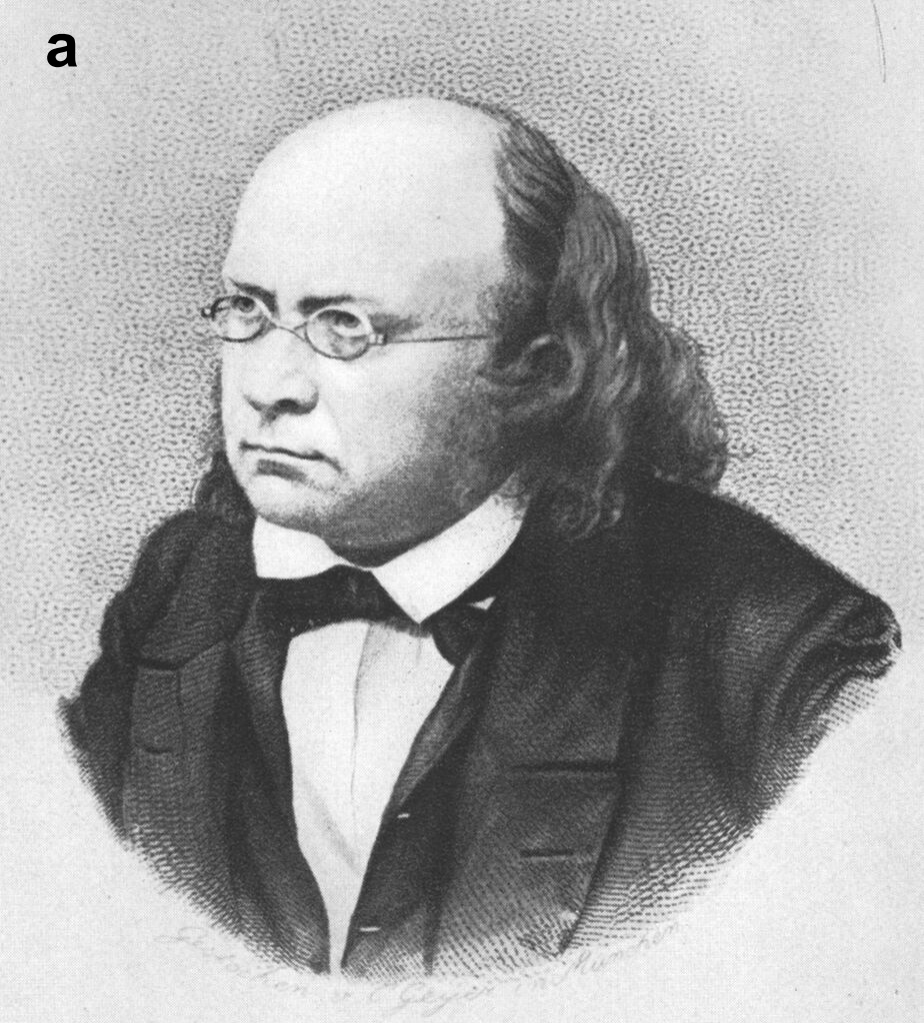
Karl Friedrich Schimper, by Conrad Geyer (1816-1893), public domain, via Wikimedia Commons

**Figure 8b.**
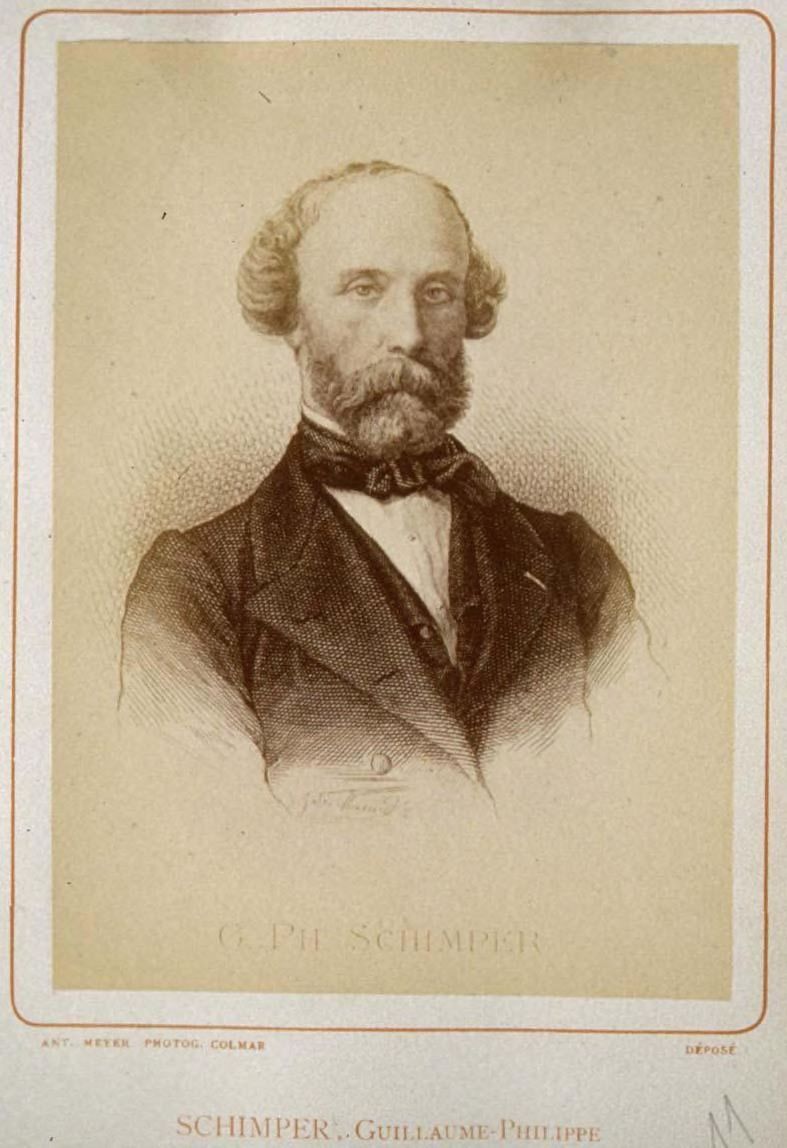
Georg Wilhelm Heinrich Schimper, public domain from the Bibliothèque nationale et universitaire de Strasbourg

**Figure 8c.**
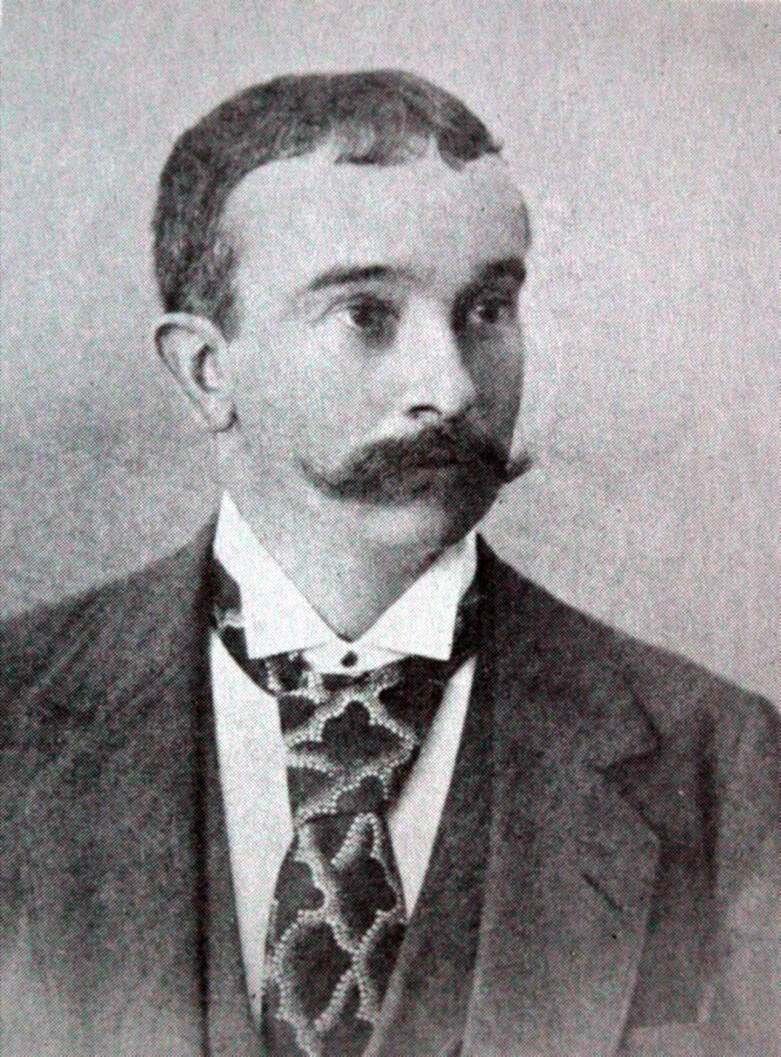
Andreas Franz Wilhelm Schimper, public domain, via Wikimedia Commons

**Figure 10a.**
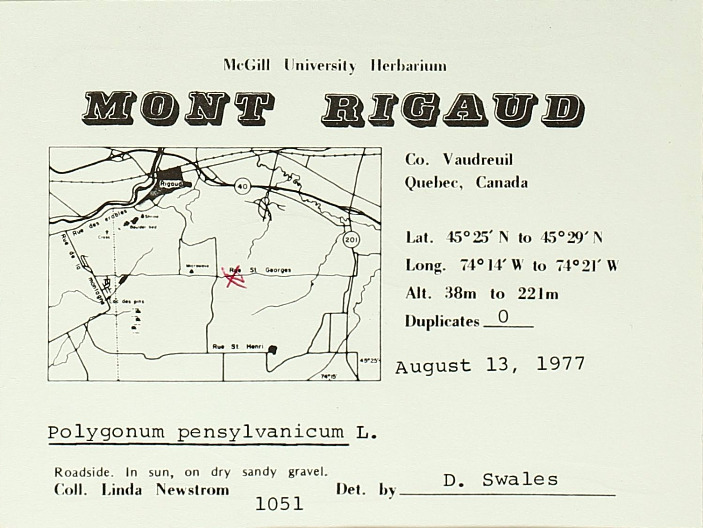
A specimen determined by Dorothy Swales as "D. Swales" from McGill University Herbarium, Catalogue number 107334

**Figure 10b.**
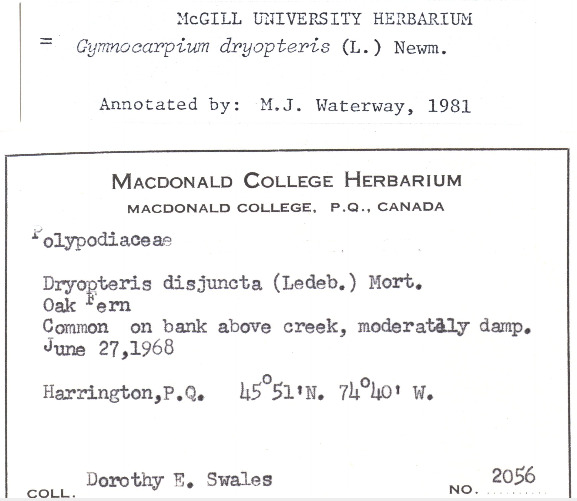
The label of a specimen collected by Dorothy Swales as "Dorothy E. Swales" from McGill University Herbarium, Catalogue number 65086.

**Figure 10c.**
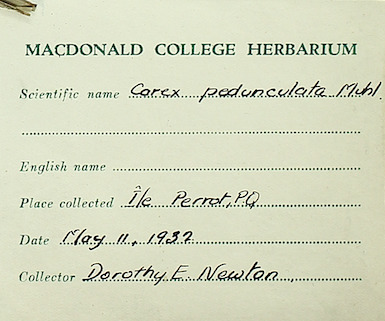
A label of a specimen collected by Dorothy Swales as "Dorothy E. Newton" from McGill University Herbarium, Catalogue number 69909.

**Figure 10d.**
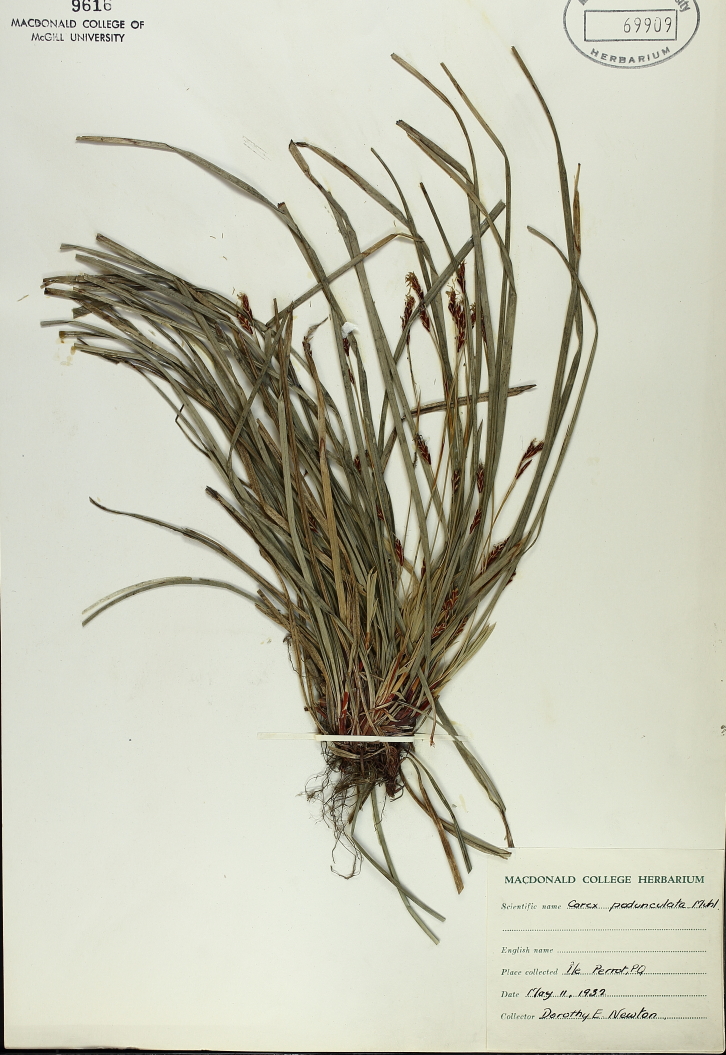
The whole specimen collected by Dorothy Swales as "Dorothy E. Newton" from McGill University Herbarium, Catalogue number 69909.
