# Supplementary material for: The disambiguation of people names in biological collections
Source: Biodivers Data J. 2022 Oct 10;10:e86089. doi: 10.3897/BDJ.10.e86089 (PMC9836581; doi:10.3897/BDJ.10.e86089)
Supplement: Supplementary material 1 — A disambiguation strategy [file bdj-10-e86089-s001.pdf]

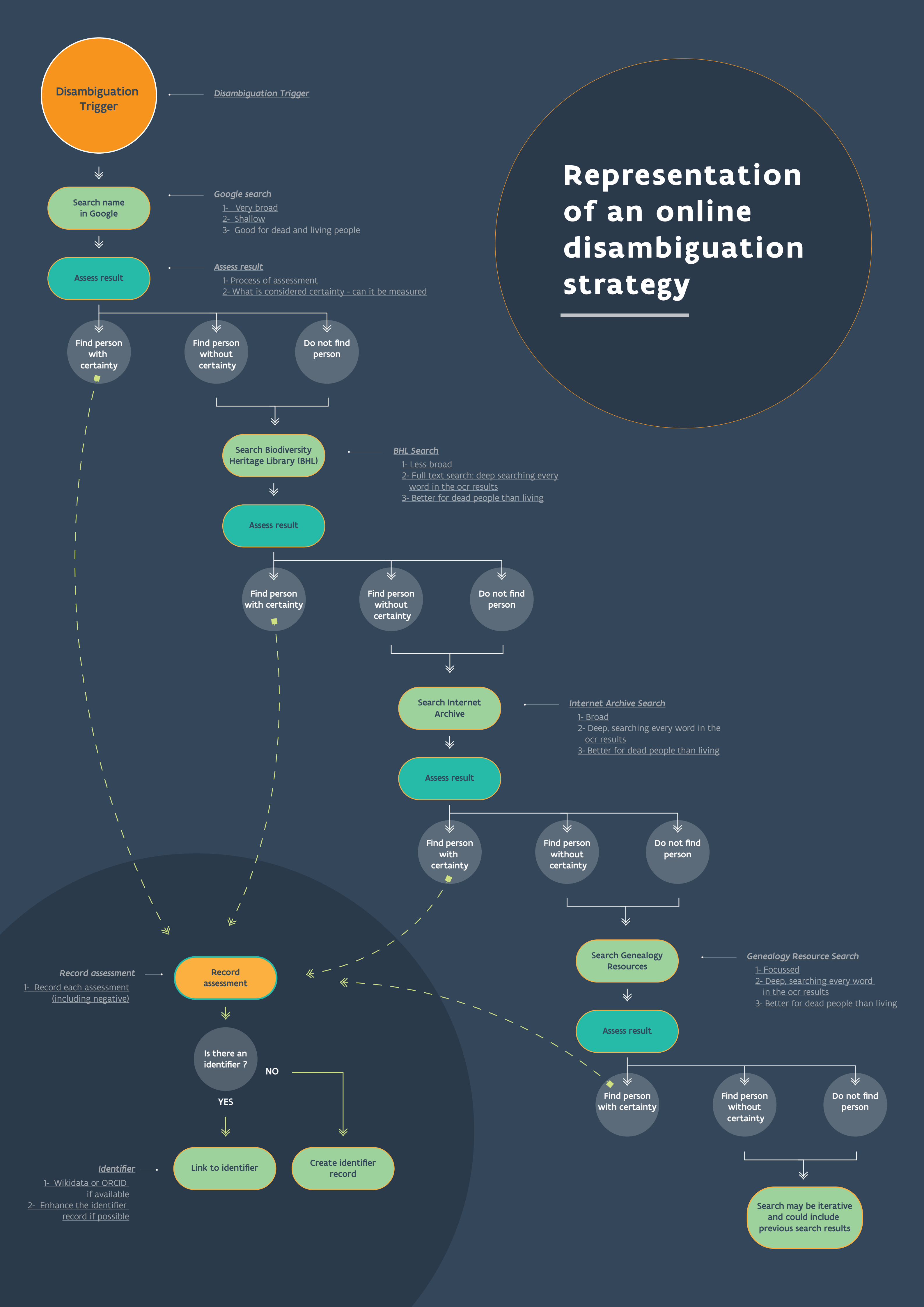

Representation of an online disambiguation strategy
Disambiguation Trigger
Disambiguation Trigger
Search name in Google
Google search
1- Very broad
2- Shallow
3- Good for dead and living people
Assess result
Assess result
1- Process of assessment
2- What is considered certainty - can it be measured
Find person with certainty
Find person without certainty
Do not find person
Search Biodiversity Heritage Library (BHL)
BHL Search
1- Less broad
2- Full text search: deep searching every word in the ocr results
3- Better for dead people than living
Assess result
Find person with certainty
Find person without certainty
Do not find person
Search Internet Archive
Internet Archive Search
1- Broad
2- Deep, searching every word in the ocr results
3- Better for dead people than living
Assess result
Find person with certainty
Find person without certainty
Do not find person
Search Genealogy Resources
Genealogy Resource Search
1- Focussed
2- Deep, searching every word in the ocr results
3- Better for dead people than living
Assess result
Find person with certainty
Find person without certainty
Do not find person
Search may be iterative and could include previous search results
Record assessment
Record assessment
1- Record each assessment (including negative)
Is there an identifier ?
NO
YES
Link to identifier
Create identifier record
Identifier
1- Wikidata or ORCID if available
2- Enhance the identifier record if possible
