# Supplementary figures and images for: The disambiguation of people names in biological collections
Source: Biodivers Data J. 2022 Oct 10;10:e86089. doi: 10.3897/BDJ.10.e86089 (PMC9836581; doi:10.3897/BDJ.10.e86089)

# Real example of a herbarium collection disambiguation process

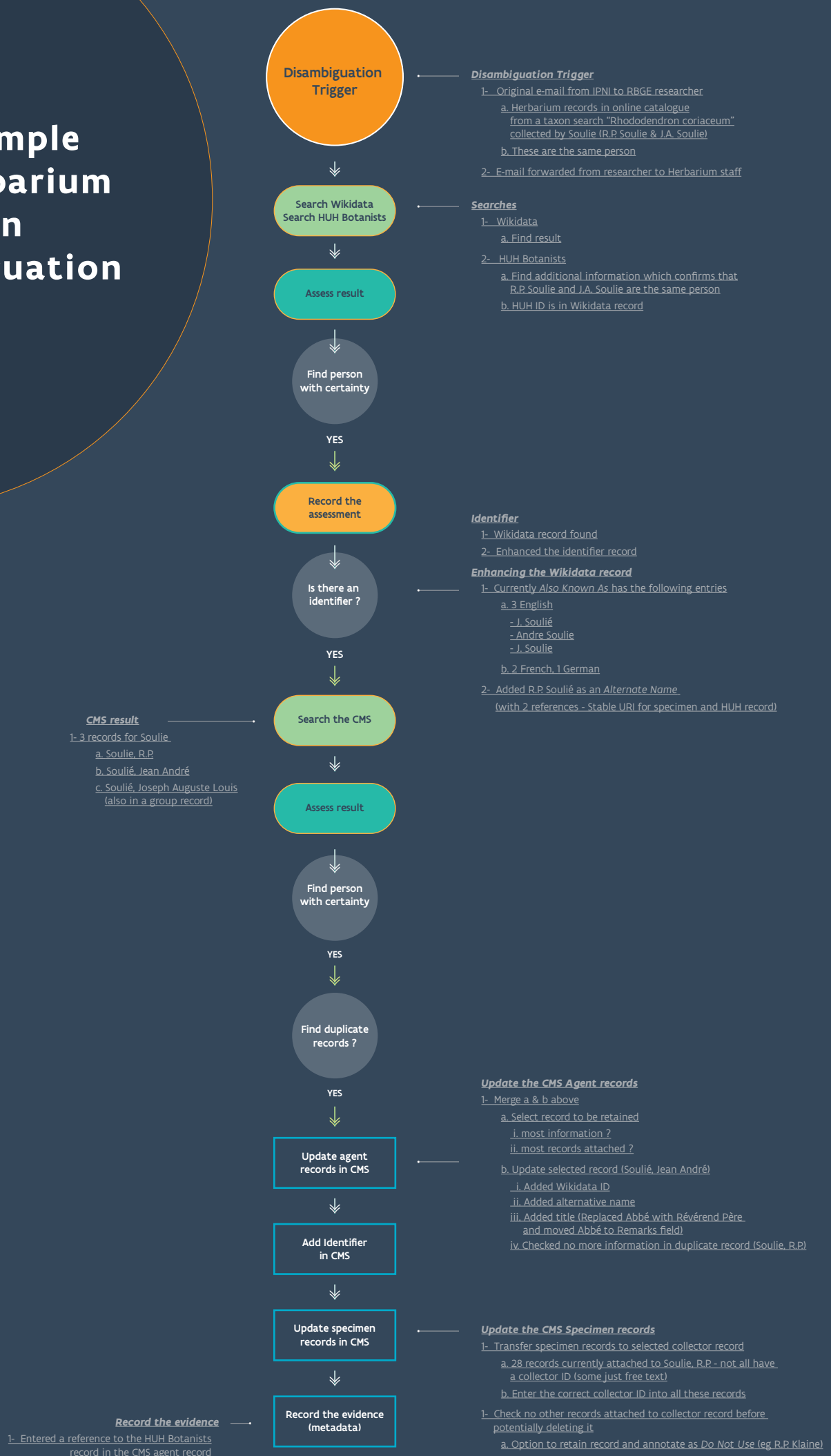

Supplement: Supplementary material 2 — Disambiguation flow example [file bdj-10-e86089-s002.pdf]
